# 

**Published:** 1917-10-27

**Authors:** 


### CONTENTS.

| | PAGE |
|---|---|
| **Memorials to Hospital Worthies** ... ... ... | 61 |
| **War Questions—Payments for Hospital Patients** | 62 |
| **Hospital and Institutional News** ... ... . | 63 |
| The Medical War Museum—Ministry of Health Bill Shelved—Simplification of National Insurance Administration—The Elimination of Typhoid—The Canadian Hospitals in France—The National Medical Union—New Proposals for Disabled Soldiers—Soldiers and Asylums—The Welfare of the Soldier—The Norfolk Hospital and the Jenny Lind Infirmary—Underfed Staffs in Infirmaries—Memorial Beds at Cardiff—Mr. Rea's Manifesto—Three-Ha'pence a Week—Red Cross Poster Stamps—The Stockport Extension Scheme near Completion—A Hospital and the War Losses Commission—Mr. Lloyd George's Thanks to the Staffs—The Term of Office of Absent Honoraries—Chaplains and Ambulance-Men—The Jack Cornwell Memorial Cot—The Spare Bedroom—Nurse-Midwives for Derbyshire—The Lack of Midwives in Cornwall—Imaginative Soldier-Patients—Light-weight Splints—School Dental Clinics—No £100 a Year Massage Assistants—The Watering of Milk—This Week's Drug Market—To Our Readers. | |
| **Health and the State** ... ... ... ... ... ... | 69 |
| A Plea for Action Based on Knowledge. | |
| **Report of the Medical Department of the Board of Education** ... ... ... ... | 71 |
| **Tuberculosis Topics** ... ... ... ... ... | 72 |
| **The Editor's Letter-Box** ... ... ... ... | 73 |
| "Raising a Ghost it Were Better to Lay"—The Irish Board of the College of Nursing. | |
| **Nursing Affairs in Ireland** ... ... ... ... ... | 74 |
| **The Matrons' and Sisters' Department** ... | 75 |
| British Nursing Advancing: What "British Women's Hospital" Stands For. | |
| Round the Hospitals. | |
| One Million for "Our Day"—Splendid Courage of British Nurses—A Thankoffering from the British Empire—Miss Rundle and the R.B.N.A.—The Beautiful Medals of St. Thomas's—Names of the Winners. | |
| **Some Hospital Types of British Nursing** ... | 78 |
| Sister B., a Memory. | |
| **The British Hospitals Association** ... ... | 79 |
| Hospital Treatment for Wounded and Pensioned Soldiers. | |
| **Matrons' and Sisters' Appointments** ... ... | 84 |
| **The Book Market** ... ... ... ... ... ... | 84 |
| **Parliament and the Profession** ... ... ... | 84 |
| **The Institutional Worker** ... ... (Suppl.) | 1 |
| Starting a Venereal Block. | |
| Enquiries and Answers. | |
| Employment and Training. | |
| The Housekeepers' Department. | |
| Miscellaneous. | |

